# Co-Occurrence Patterns of Plants and Soil Bacteria in the High-Alpine Subnival Zone Track Environmental Harshness

**DOI:** 10.3389/fmicb.2012.00347

**Published:** 2012-10-11

**Authors:** Andrew J. King, Emily C. Farrer, Katharine N. Suding, Steven K. Schmidt

**Affiliations:** ^1^Ecosystem Sciences, Commonwealth Scientific and Industrial Research OrganisationActon, ACT, Australia; ^2^Department of Environmental Science, Policy & Management, University of California at BerkeleyBerkeley, CA, USA; ^3^Department of Ecology and Evolutionary Biology, University of Colorado at BoulderBoulder, CO, USA

**Keywords:** community assembly, co-occurrence networks, facilitation, mutualism, Niwot Ridge, plant-microbe interactions, soil microbial communities, symbiosis

## Abstract

Plants and soil microorganisms interact to play a central role in ecosystem functioning. To determine the potential importance of biotic interactions in shaping the distributions of these organisms in a high-alpine subnival landscape, we examine co-occurrence patterns between plant species and bulk soil bacteria abundances. In this context, a co-occurrence relationship reflects a combination of several assembly processes: that both parties can disperse to the site, that they can survive the abiotic environmental conditions, and that interactions between the biota either facilitate survival or allow for coexistence. Across the entire landscape, 31% of the bacterial sequences in this dataset were significantly correlated to the abundance distribution of one or more plant species. These sequences fell into 14 clades, 6 of which are related to bacteria that are known to form symbioses with plants in other systems. Abundant plant species were more likely to have significant as well as stronger correlations with bacteria and these patterns were more prevalent in lower altitude sites. Conversely, correlations between plant species abundances and bacterial relative abundances were less frequent in sites near the snowline. Thus, plant-bacteria associations became more common as environmental conditions became less harsh and plants became more abundant. This pattern in co-occurrence strength and frequency across the subnival landscape suggests that plant-bacteria interactions are important for the success of life, both below- and above-ground, in an extreme environment.

## Introduction

Interactions between plants and microorganisms play a central role in ecosystem functioning (Bonfante and Anca, 2009; Hayat et al., 2010). However, it has been difficult to characterize plant-microbial relationships for all but the most closely interacting species (Kremer, 2006; van der Heijden et al., 2008; Hayat et al., 2010). For example, while mutualistic and parasitic symbiotes inhabiting root tissue have been well studied (mycorrhizae: Bianciotto et al., 1996; Bonfante and Anca, 2009; diseases: Compant et al., 2005; Kremer, 2006; N-fixation: Hayat et al., 2010; heat tolerance: Márquez et al., 2007; plant growth hormone production by bacteria: Hayat et al., 2010), it appears that these interactions just scratch the surface of the probable range of symbioses (Li and Kremer, 2006; Rudgers et al., 2012). Similarly, the indirect associations involved with decomposition and nutrient cycling between microorganisms and plants, despite being well studied from a biogeochemical perspective, have remained difficult to characterize with respect to the soil microbial species involved (van der Heijden et al., 2008). In addition to the difficulty imposed by frequent findings of new plant-bacteria interactions, predicting the frequency and types of known plant-soil microbe associations is often difficult even when species lists and environmental conditions are known (Bever et al., 2010). The success of targeted studies using cultures (Kremer, 2006; Hayat et al., 2010) and stable isotope probing (Haichar et al., 2008) to identify plant-soil interactions suggests that larger scale surveys using high-throughput methods may allow for the detection of broad patterns for plant-soil microbe interactions from bulk soil. Here, for the first time, we use culture-independent methods to test hypotheses regarding the drivers of co-occurrence relationships between plants and soil bacteria in bulk soil samples and at the landscape scale.

The co-occurrence of species that share resource requirements is governed by competitive (Diamond, 1975) and facilitative (Bruno et al., 2003) interactions between species as well as independent environmental sorting in the case of strong environmental filters (Weiher and Keddy, 1995; Diaz et al., 1998; Ackerly, 2003). As such, co-occurrence patterns represent an important tool for inferring potential interactions involving microorganisms, particularly bacteria, which are most commonly identified indirectly via isolation of organisms or sequencing their DNA from soil extracts (Ruan et al., 2006; Horner-Devine et al., 2007; Fuhrman and Steele, 2008; Freilich et al., 2010; Barberán et al., 2012). Because the methods enabling the analysis of high-throughput, sequencing based, broad-scale surveys of bacteria are very new (Caporaso et al., 2011; Gonzalez et al., 2011), the frequency and strength of plant-bacteria co-occurrence relationships at a landscape scale are poorly understood. Thus, high-throughput sequencing of bulk soil samples represents a potentially powerful resource for the detection of plant-bacteria interactions such as saprotrophic bacteria that specialize on senesced material from a particular plant species, bacteria sharing similar environmental preferences, and symbiotic rhizosphere bacteria which are often found to be a well represented subset of bulk soil samples (Mahaffee and Kloepper, 1997; Graff and Conrad, 2006; Berg and Smalla, 2009).

We examine plant-bacterial co-occurrence relationships along an abiotic harshness gradient in a high-alpine subnival ecosystem because the resulting gradient in plant abundance represents increasing availability of reduced carbon for soil microorganisms (Ley et al., 2004) and the oligotrophic soils mean plant symbioses with soil microorganisms are particularly important (Chapin et al., 1994; Tscherko et al., 2005). The harsh environmental conditions of the subnival zone result from high altitudes with their concurrent high solar radiation, low humidity, large daily temperature fluctuations that often cross the freezing point, large snowpack volumes or high wind exposure, and low soil nutrients (Ley et al., 2004; Freeman et al., 2009; King et al., 2010). Thus, subnival organisms must persist in a state of strong environmental stress which prohibits continuous plant cover, but is not extreme enough that there is year-round snow or ice cover. Previous studies in slightly less harsh, alpine ecosystems have shown that the majority of plants are able to form mutualistic associations with fungi, N-fixing bacteria, and growth promoting bacteria (Mullen et al., 1998; Cazares et al., 2005; Sheng et al., 2010), suggesting that mutualistic associations are important for the survival of plants and bacteria in these systems (Hobbie et al., 2005; Wang and Qiu, 2006; Schmidt et al., 2008a; Sheng et al., 2010; Zinger et al., 2011). However, in subnival glacial fore-fronts these same well-colonized plant species often lack root mutualistic symbionts and this lack of symbionts is thought to be due to dispersal limitation of the microorganisms in the newly exposed substrate (Cazares et al., 2005). Pathogens of alpine plants, on the other hand, are rarely reported (Olofsson et al., 2011); it may be that pathogens are rare or it could be that few studies explicitly focus on this group. Thus, positive plant-bacterial interactions are of high potential importance in subnival landscapes.

We draw from the body of theory regarding community assembly (Diamond, 1975; Chapin et al., 1994; Chase and Leibold, 2003; Reynolds et al., 2003) to develop simple yet fundamental hypotheses about the drivers of the frequency and distribution of plant-bacteria co-occurrence relationships. A basic tenet of community assembly theory is that abiotic and biotic “filters” select species from the regional species pool to assemble a local community most suited to the prevailing conditions in a process known as ecological sorting (Weiher and Keddy, 1995; Diaz et al., 1998; Ackerly, 2003). In the context of plant-bacteria co-occurrence in the alpine subnival environment, we expect the presence of biotic filters that restrict one group to occur only when its symbiont/facilitator is present as well as a common set of abiotic and dispersal filters that may constrain both plants and microbes directly. Biotic filters may be reflected by bacteria with plant growth promoting/inhibiting abilities or plants that promote the growth of mutualistic soil bacteria. If certain bacteria and plants have similar environmental preferences, they may co-occur across space independently without directly interacting with each other. For soil bacteria that are, in fact, interacting with plant species, negative correlations can arise from inhibitory and competitive interaction whereas positive correlations can arise from facilitative, mutualistic, or parasitic interactions.

Based on this community assembly framework, we explicitly test three hypotheses about the drivers of the frequency and distribution of plant-bacteria co-occurrence relationships. First, analogous to the suitable habitat area-diversity relationship from island biogeography (MacArthur and Wilson, 1967; Martiny et al., 2006), we expect bacteria to more often co-occur with plant species’ that provide larger and more consistent nutrient sources across the landscape. Assuming that a plant species contribution to nutrient pools is proportional to its abundance (Grime, 1998; Vile et al., 2006), we thus predict that abundant plants will have more bacterial clades associated with them than less abundant plants.

Second, we expect that bacterial clades related to isolates with plant growth promoting/inhibiting abilities will show stronger species-specific associations with plants than will bacteria related to isolates with plant-independent metabolic strategies. Thus, the hypothetical functional capacity for associating with a plant species represents a biotic filter (e.g., Chase and Leibold, 2003) for the occurrence of bacteria in the subnival landscape. Importantly, evaluation of this driver is also testing the utility of a correlation approach using culture-independent environmental sequencing of the 16S to identify clades that have a specific function.

Lastly, we predict that facilitation should be more common in portions of the landscape furthest from intact tundra due to increasing environmental harshness. Importantly, theory predicts that facilitative and mutualistic plant-microbe interactions are more common in harsher, low density, low nutrient availability, successional environments (Reynolds et al., 2003) because they promote the growth of both bacteria and plants. Such facilitative interactions are thought to be able to overcome environmental filters that would otherwise exclude either symbiont (Choler et al., 2001). Although it is likely endemic extremophiles are adapted to the harsh environment of the subnival zone, our previous work has shown that the ancestry of bacteria does not change significantly across the landscape (King et al., 2010). Therefore, we expect that the lower temperatures, greater UV radiation, and less soil nutrients with increasing remoteness (Körner, 2007) represent real increases in stress to bacteria in this landscape.

## Materials and Methods

### Study site

**Figure 1:**
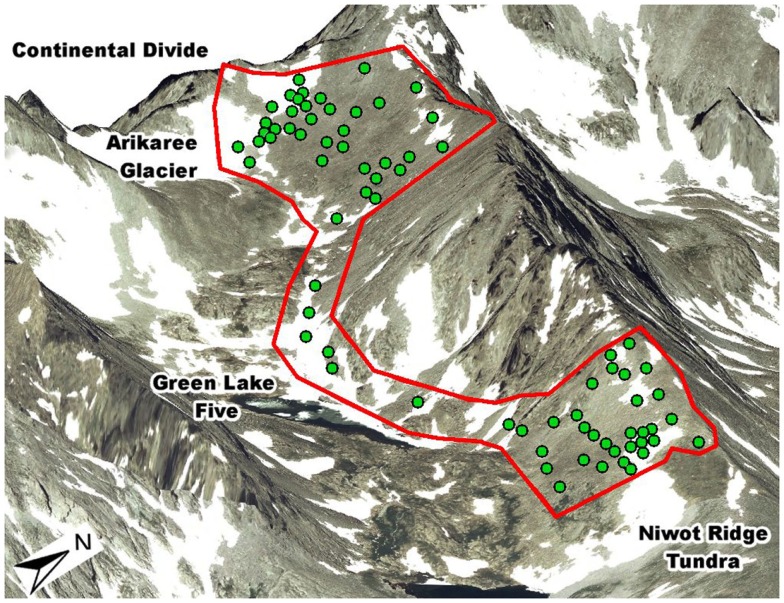
**Our 76 sample sites in the subnvial zone of the Green Lakes Valley within the Niwot Ridge LTER study site on eastern face of the Continental Divide in Colorado, USA (red outline represents the south-facing extent of exposed soils)**. From the north-east corner to the north-west corner of the study area is 2 km. These sites represent a subset of the sites in our previous study (shown in Figure 1 of King et al., 2010).

The dataset used in this analysis comes from a previously published spatially explicit study of the Niwot Ridge LTER’s south-facing subnival slope (eastern face of the Continental Divide in Colorado, USA; Figure 1; King et al., 2010). This slope is covered with snow from October to June and the deepest snow fields do not melt fully until August. Precipitation averages 930 mm/year with 80% falling as snow (Nemergut et al., 2005). The study area extends over 2 km^2^ and is predominately covered by granite blocks greater than 1m in diameter. Interspersed are patches of soil up to 20 m in diameter with plant cover ranging from 0% in late melting snowbanks up to 100% in a few exposed areas receiving water from upslope snowmelt. Plants in this environment are 10–20 cm in height and have approximately the same biomass per individual.

### Plant species and soil sampling

As previously reported (King et al., 2010), sampling locations were chosen based on a pilot study (King et al., 2008) in order to provide optimal spacing for the calculation of spatial autocorrelation models across the landscape. Sampling was partitioned into a grid of 160 samples across the landscape, spaced every 50 m, with three targeted 30 m × 30 m areas sampled at every 5 m (Figure 1 in King et al., 2010). In September of 2007, at each sampling location a pen was tossed into the air and at the spot where it landed all vascular plants within a 3 m^2^ circle were identified and a soil sample was collected. Plant abundance measures were based on stem counts. Because alpine species are constrained to a low stature growth form, stem counts are an appropriate estimate of species biomass within a plot (Grytnes, 2000). Species identification was based on Webber and Whittmann (2001).

Soils samples were collected by homogenizing a ∼50 cm^2^ patch of soil *in situ* to a depth of 4 cm and then filling a 50 mL sterile conical tube with soil. Because of the high degree of spatial autocorrelation in bacterial community diversity in this system, an individual sample is likely to be highly representative of the surrounding 25 m^2^ of soil (King et al., 2010). Samples were transported to the lab within 5 h of collection, held at 4°C for up to a week while subsampling for soil biogeochemistry was conducted (results presented in King et al., 2010), and afterward frozen at −20°C until DNA could be extracted (up to 9 months). 85 of the original 160 samples were randomly selected for bacterial community analysis. From those 85 samples, we include in the current study only plots that had at least two plants per m^2^ (76 samples, Figure 1).

### Bacterial community assessment

Bacterial community composition was also measured as described in King et al. (2010). Briefly, for each of the 76 soil samples, DNA was extracted from 1 g of soil using a Mobio PowerSoil DNA Isolation Kit (Mobio Laboratories) and PCR was used to amplify the V1–V2 hypervariable region of the bacterial 16S SSU ribosome gene using the 27F and 338R primers and protocol from Fierer et al. (2008). Sequencing was performed on the Roche 454 platform using FLX chemistry. Raw sequence data was processed using the methods of Hamady et al. (2010), resulting in 6151 representative OTUs at 3% similarity with 10,000 total reads and an average of 200 reads per sample. For downstream analysis, sequence reads of each OTU in each sample were converted to relative abundance by dividing by the total number of sequences in an individual sample.

### Selection of clades using correlation strength with plant species abundances

Because most methods of assigning taxonomic IDs to individual sequences are based on the extent of knowledge contained in a reference database (e.g., DeSantis et al., 2006; Wang et al., 2007) and because classical taxonomies can be polyphyletic, we used a phylogenetic tree to assign sequences to clades for downstream analysis (the phylogenetic tree was the same as used in King et al. (2010)). This approach is essentially a phylogenetic comparative method (Harvey and Pagel, 1991) with association with plants as the trait of interest. Initially we considered as candidate clades all nodes on the tree that had greater than 1% of the sequences (therefore, clades are independent of phylogenetic resolution and a particular clade might correspond to any gradation between phylum and species). Each candidate clade’s relative abundance per sample was then regressed against the abundances of the 13 most prevalent plant species (all plant species averaging greater than one individual per sampling location; the total plant species pool for this landscape is 26). Regressions were performed in R using the Akaike Information Criterion (Akaike, 1974) to select the plant species with the most explanatory power via the step and lm functions (R Development Core Team, 2011). A strictly linear approach was chosen to maintain a low number of parameters and residuals from both the models, and pairwise regressions of clades against individual plant species were found to be normally distributed.

The initial list of clades was then filtered so that clades were mutually exclusive; this filtering was achieved via sorting the clades by *r*^2^, removing all clades that had a lower *r*^2^ but shared sequences with a clade that had the highest *r*^2^, and then reiterating with the next highest remaining *r*^2^ until reaching a clade without a significant correlation or an *r*^2^ less than 0.15. The remaining 22 clades were tested for significance with an ANOVA with the AIC selected variables as explanatory variables and significance cutoff adjusted for multiple comparisons using the Bonferroni correction for *n* = 22 comparisons (*p* < 0.002; Bonferroni, 1936). The final result was 14 bacterial clades. Taxonomic IDs were then assigned as the finest resolution at which at least 50% of the sequences shared the same classification in their closest match in NCBI GenBank.

### Plant-bacteria co-occurrence ordination

To visualize the correlations between plant species and the bacterial clades identified by the above regressions we used a NMDS approach. First, we constructed a correlation matrix with all possible pairwise comparisons between both bacteria clades and plant species. We then used a common approach (Cox and Cox, 1994) to transform each Pearson correlation value (ranging from 1 to −1) by taking the absolute value and subtracting it from one such that both positive and negative 100% correlations became a distance of 0 and a 0% correlation became a distance of one. The resulting distance matrix was used as an input to the metaMDS function in the vegan package for R (Oksanen et al., 2010). While co-occurrence networks in microbiology have most commonly been arranged so that the nodes with the greatest numbers of edges are in the center of the plot (Barberán et al., 2012), NMDS is very commonly used in plant ecology to represent strength (rather than number) of interactions (McCune et al., 2002). We chose NMDS so that the strength of interaction between each clade or plant species in the dataset would be represented by proximity. In addition, we used NMDS to ordinate our plant-bacteria co-occurrence relationships rather than the more common metric MDS approach (Cox and Cox, 1994) because NMDS gives greater weight to the strongest correlations (Cox and Cox, 1994) and relatively little weight to weaker correlations (MDS techniques would have used the raw correlation value and the large number of non-significant correlations with values of 0.20 or less would have introduced extra noise into the ordination). After the species and clades were ordinated, we visually overlaid the significant interactions according to our models.

### Statistical analysis

To quantitatively estimate the average strength of association for a particular plant species or bacterial clade, we averaged the individual correlation *r* values (absolute value) that were significant according to our AIC supervised linear models. (*p* < 0.05, two-tailed *t*-test for each regression coefficient not equal to 0). Bonferroni correction of the *p*-value for multiple comparisons was done for each model’s coefficient significance tests (clade relative abundance models ranged from one to eight AIC selected plant species, Supplementary Materials).

To address the hypothesis that more abundant plants are better predictors of the abundance of associated bacterial clades, the number of significantly correlated clades per plant species was regressed against plant species abundance and we used a *t*-test to determine if the correlation was significantly greater than zero. The mean correlation strength of clades associated with each plant species was also regressed against plant species abundance and we used a *t*-test to determine if the correlation was significantly greater than zero. These tests were performed both including and excluding plant species with no significantly correlated bacterial clades.

To address the hypothesis that average association strength would be greatest for bacterial clades closely related to bacterial species that are known to form symbioses with plants, we performed a *t*-test comparing mean correlation strengths of each model’s significant factors for clades related to plant symbiosis capable versus plant-independent bacteria [i.e., Avg Corr Str (*r*) column of Table 1].

**Table 1:** **A summary of known metabolic capabilities, OTU richness, number of significant plant-associations, and model fits for plant-associated subnival zone bacterial clades**.

Clade	Plant symbiosis	Symbiosis location	Free-living metabolism	Reference	Average abundance (SD)	OTU richness	Positive interactions	Negative interactions	Model *r*^2^	Avg corr str (*r*)	Harsh environ up *r*^2^	Corr number harsh env up	Avg corr harsh up (*r*)	Harsh env down *r*^2^	Corr number harsh env down	Avg corr harsh down (r)
Acidimicrobiaceae	Unknown		S-oxidation, Fe oxidation, N-fixation chemotrophy	Buckley et al. (2007), Norris et al. (2011)	1.11 (1.1)	58	1	0	0.26	0.41	0.29*	2	0.37	0.34*	2	0.27
Acidobacteria_Gp1	Unknown		Unknown	Lee et al. (2006)	2.76 (2.2)	84	1	1	0.34	0.29	0.21*	2	0.26	0.32*	1	0.51
Acidobacteria_Gp3	Unknown		Unknown	Lee et al. (2006)	1.18 (1.4)	63	1	0	0.26	0.3	0.27*	1	0.28	0.3*	2	0.32
Acidobacteria_Gp4	Unknown		Unknown	Lee et al. (2006)	1.22 (1.5)	67	1	0	0.32	0.51	0.13	0		0.42*	1	0.63
Acidobacteria_Gp7	Unknown		Unknown	Lee et al. (2006)	2.77 (2.7)	67	0	1	0.24	0.31	0.08	0		0.16*	1	0.14
Burkholderiales	N-fixation, P-mobilization pathogenic	Intra/ extracellular roots stems	Heterotrophy endosymbiont	Rodrigues-Diaz et al. (2008), Compant et al. (2008)	1.47 (1.7)	54	1	0	0.4	0.52	0	0		0.41*	2	0.32
Clostridiales	Growth promoting pathogenic	Extracellular	Heterotrophy, N-fixation	Rodrigues-Diaz et al. (2008)	1.32 (1.8)	3	2	0	0.38	0.46	0.07	0		0.69*	3	0.55
Deltaproteobacteria	Unknown		Heterotrophy, S-reduction, Fe-reduction	Brenner et al. (2005)	5.01 (2.7)	503	1	0	0.24	0.26	0.27*	2	0.26	0.3*	2	0.24
Desulfovibrionales	Nematicidal	Extracellular endophytic roots	Heterotrophy, S-reduction	Rodrigues-Diaz et al. (2008)	1.30 (1.1)	153	0	2	0.24	0.26	0.12	0		0.34*	3	0.17
Ktedonobacteraceae	Unknown		CO-oxidation	Webber and King (2010)	5.12 (5.4)	320	1	0	0.28	0.31	0.26*	1	0.51	0.32*	2	0.42
Pseudonocardiaceae	N-fixation	Endophytic roots	Heterotrophy, S-oxidation, N-fixation	Reichert et al. (1998), Chen et al. (2009)	1.14 (1.7)	24	1	0	0.24	0.3	0.35*	2	0.44	0.18	0	
Rhizobiales	N-fixation, pathogenic	Extracellular endophytic	Heterotrophy, N-fixation	Rodrigues-Diaz et al. (2008), Carvalho et al. (2010)	3.28 (2.3)	205	1	0	0.22	0.3	0.2	0		0.47*	2	0.35
Rhodospirillales	N-fixation, P-mobilization	Extracellular endophytic leaves, stems, roots	Heterotrophy, phototrophy, N-fixation, S-reduction	Madigan (2005), Rodrigues-Diaz et al. (2008)	2.25 (2.4)	119	2	0	0.31	0.21	0.44*	2	0.47	0.2	0	
TM7	Unknown		Unknown	Marcy et al. (2007)	0.96 (1.2)	86	1	0	0.3	0.39	0.24	0		0.33*	1	0.55

*Indicates a significant model fit with harshness up- or harshness down-weighted data, ANOVA, *p* < 0.004.

To address the hypothesis that environmental harshness affects association strength, we reweighted all already identified sets of bacterial clade relative abundances by a harshness index (a proxy for the subnival landscape’s general gradient of increasing exposure, temperature variability, and time before complete snowmelt with increasing distance away from intact tundra). The environmental harshness index for each sample was calculated as distance from intact tundra + distance from valley floor. In addition to distance from tundra and altitude, the degree of shelteredness from wind is also expected to play a role in structuring species co-occurrence in this subnival landscape (Litaor et al., 2008). However, although both high and low snow accumulation areas vary in the nature of the primary stressor (late snowmelt versus high wind exposure), snowfields and windswept areas occur throughout our study area (Erickson et al., 2005). Due to this variability, our harshness metric is not correlated with snow depth (Correlation test, *p* = 0.2, *r*^2^ = 0.02) in the Niwot subnival zone, suggesting that harshness overlays a matrix of sheltered and unsheltered areas which occur throughout the landscape. Thus, our proposed harshness metric is similar in nature to altitudinal gradient analysis in that it is strongly correlated with gradients in temperature/solar insolation that overlay landscape heterogeneity in shelteredness (Körner, 2007).

We examined the effect of multiplying abundances by the harshness index (up-weighting harshness). We also examined the effect of multiplying abundances by the harshness subtracted from the max harshness value (down-weighting harshness). For up- and down-weighted datasets we recalculated linear model fits for each of the previously identified bacterial clades using AIC selection of significantly predictive plant species. The effect size of reweighting was then calculated by averaging the individual correlation *r* values (absolute value) between plant species and bacterial clades that were significant first for the upweighted and then for the down-weighted datasets. To test if a significant effect of up- or down-weighting was observed for a specific clade, we used an ANOVA with the AIC selected variables as explanatory variables and significance cutoff adjusted for multiple comparisons using the Bonferroni correction for *n* = 14 comparisons (*p* < 0.004; Bonferroni, 1936). Again, the clade average correlation strengths reflect only significant correlations after Bonferroni correction for each model’s set of AIC selected variables.

## Results

### General findings

**Figure 2:**
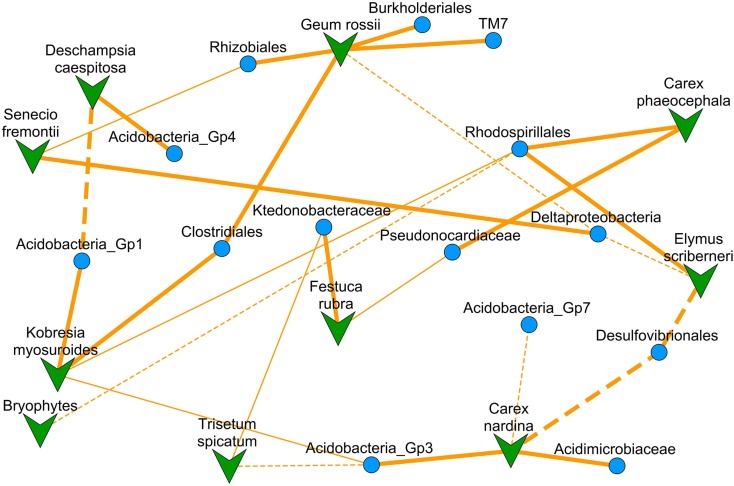
**A NMDS (stress = 0.292) ordinated co-occurrence network based on the correlation matrix (absolute values of Pearson *r*) for 10 plant species abundances and 14 bacterial clade relative abundances (Datasheet S4 in Supplementary Material)**. Overlaid are lines designating significant model-based interactions between a plant species and bacterial clade with solid for positive and dashed for negative interactions (thin lines represent two-tailed *t*-test for model coefficient significance, *p* < 0.05; thick lines represent significance after Bonferroni correction for multiple comparisons). Because this is an ordination of the correlation matrix, proximity of points to one another represents higher correlation between their abundances across the subnival landscape.

**Figure 3:**
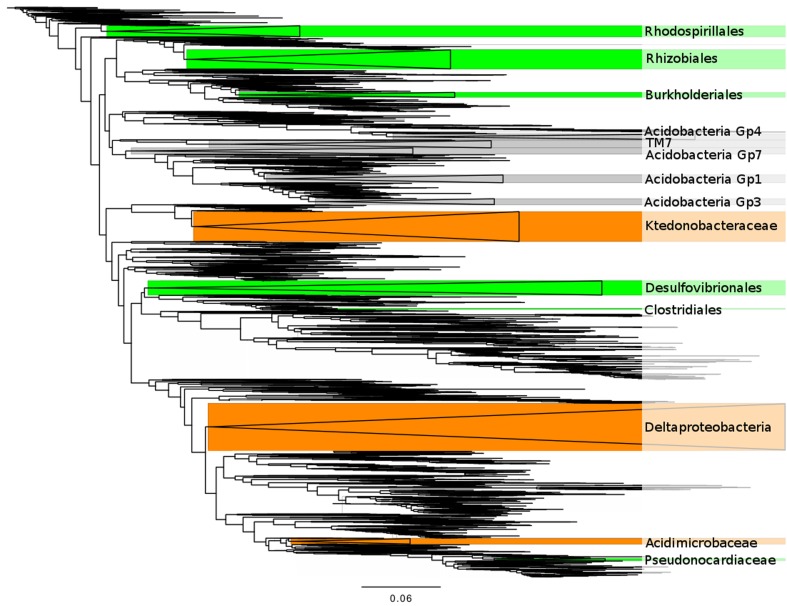
**A phylogenetic tree showing the clades correlated with plant abundance. Green clades are commonly found associated with plants in the scientific literature (Table 1)**. Orange clades have no known symbiotic association with plants. Gray clades are only known from environmental sequencing. Wedge size is proportional to the number of 3% OTU clusters in a clade.

The NMDS ordinated co-occurrence network contained 14 bacterial clades, representing 31% of the total sequences in our study, and 10 of the 13 plant species (Figure 2). Significant correlations were not dependent on the diversity of bacterial OTUs, total branch length within the clade, or relative abundance of clades (Figure 3). The percentage of variance in clade relative abundance significantly explained by plant species abundance ranged from 40% to our minimum cutoff of 15% (Table 1). Negative correlations, at 3, were less common than positive correlations, at 14 (Figure 2; Table 1). Bacterial clades had one or two significantly correlated plant species; only one had exclusively negative significant correlations with plant species (Desulfovibrionales), and two had multiple positive associations (Clostridiales and Rhodospirillales). Our models predicting bacterial clade relative abundance based on plant species abundance with 13 potential explanatory plant species (Datasheet S1 in Supplementary Material) only slightly under-perform compared to previously published environmental models for the three most spatially structured clades examined by King et al. (2010) with 21 potential explanatory variables; on average our models explain 28% of variance in clade relative abundance for Rhodospirillales, Rhizobiales, and Acidobacteria Gp4, compared to 40% for the previously published (King et al., 2010) models using plant abundance, soil biogeochemistry, and spatial autocorrelation as predictors.

Six of the 14 bacterial clades (comprising 10% of the total sequences) are related to isolates that are known for forming symbioses with plants (Table 1) for N-fixation and/or plant growth promotion: Rhizobiales, Rhodospirillales, Clostridiales, Desulfovibrionales, Pseudonocardiaceae, and Burkholderiales (also pathogenic) in addition to their capacity of free-living heterotrophic functions (Table 1). Many of these clades are also related to isolates known to be able to perform rhizosphere-independent soil functions such as sulfur reduction (Rhodospirillales, Desulfovibrionales) and sulfite oxidation (Clostridiales, Pseudonocardiaceae). Two of the clades (comprising 9% of the sequences) lacked support for plant symbiosis capability and displayed a diverse array of metabolic strategies: carbon monoxide oxidation (Ktedonobacteraceae), general heterotrophy/sulfate + iron reducing (Deltaproteobacteria). Five of the remaining six clades (comprising 11% of the total sequences) represent common soil bacteria with no cultured representatives and largely unknown functions (four clades of Acidobacteria, TM7). The last clade, Acidimicrobiaceae, is defined by their autotrophic growth by oxidizing ferrous iron, but has been hypothesized to also inhabit the rhizosphere and root tissue based on culture-independent surveys (Stafford et al., 2005; Qin et al., 2012) and may have the capacity for nitrogen fixation (Buckley et al., 2007).

#### H1: abundant plants will have more bacteria associated with them than less abundant plants because they represent the most common plant-derived nutrient source

**Figure 4:**
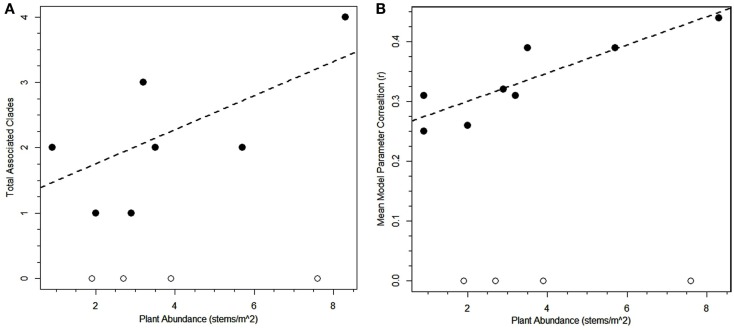
**Regressions of plant abundance versus total number of significant associated bacterial clades (A) or average strength of the correlations for associated bacterial clades (B)**. Regression lines are significant if the plant species without any significant associated bacterial clades are excluded from the analysis [*r* = 0.67, one-tailed *t*-test for rho = 0: *p* = 0.0275 **(A)**; *r* = 0.88, two-tailed *t*-test for rho = 0: *p* = 0.004 **(B)]**.

At first inspection, plant species abundance was not correlated to the number of correlated bacterial clades (*r* = 0.45, two-tailed *t*-test for rho = 0: *p* = 0.14, Table 2) or the average correlation strength for clades associated with a particular plant species (*r* = 0.25, two-tailed *t*-test for rho = 0: *p* = 0.44, Table 2). However, when plants with no correlated clades were removed from the analysis, there was a significant relationship between plant abundance and average correlation strength for clades associated with a particular plant species (*r* = 0.88, two-tailed *t*-test for rho = 0: *p* = 0.004, Figure 4B) and a weakly significant correlation for the number of correlated bacterial clades (*r* = 0.67, one-tailed *t*-test for rho = 0: *p* = 0.0275, Figure 4A). The plants with the greatest number of positive associations were *Geum rossii* (four associations), *Kobresia myosuroides* (two associations), and *Carex nardina* (two associations). Abundant plant species lacking significantly correlated bacterial clades were *Bryophytes, Trisetum spicatum, Trifolium nanum*, *Silene acaulis*.

**Table 2:** **A summary of subnival zone plant species’ abundances, number of significant bacterial clade-associations and model fits**.

Plant species	Average (plants/site)	SD	Positives	Negatives	Avg str corr (*r*)	Correlation with remote index (*r*)	Total Corr remote upweight	Avg corr rm up	Total cor remote downweight	Avg corr rm down
*Geum rossii*	8.3	24	4	0	0.44	−0.2	0	0	4	0.60
*Bryophytes*	7.6	13.7	0	0	0	0.05	1	0.52	0	0
*Deschampsia cespitosa*	5.7	14	1	1	0.39	−0.27	0	0	2	0.50
*Trisetum spicatum*	3.9	5.7	0	0	0	0.08	0	0	1	0.42
*Kobresia myosuroides*	3.5	15.3	2	0	0.39	−0.16	1	0.05	5	0.41
*Carex nardina*	3.2	5.7	2	1	0.31	−0.25	1	0.01	2	0.43
*Festuca rubra*	2.9	4	1	0	0.32	0.4	4	0.42	6	0.06
*Trifolium nanum*	2.7	10.6	0	0	0	−0.04	0	0	0	0
*Senecio fremontii*	2	6.3	1	0	0.26	−0.132	0	0	1	0.43
*Silene acaulis*	1.9	6.1	0	0	0	0.01	0	0	1	0.34
*Cirsium scopulorum*	1	2	1	1	0.25	0.05	0	0	0	0
*Elymus scribneri*	0.9	2.8	2	0	0.31	0.27	2	0.48	0	0
*Carex phaeocephala*	0.9	2.9	4	0	0.44	0.13	3	0.40	0	0

#### H2: potentially plant symbiosis capable bacterial clades will have greater average correlation strength with plant species than clades predicted to lack the capacity for symbiosis

On average, bacterial clades related to strains that were capable of forming symbioses with plants did not differ significantly in their average association strength from bacterial clades without known symbiosis capability (mean *r* was 0.34 symbiosis capable versus 0.29 incapable, two-tailed *t*-test: *p* = 0.63; Table 1). Of the bacterial clades with assignable functional capacities (Figure 3, Table 1), the Burkholderiales had the strongest association with a plant species (*G. rossii*; Figure 2; Datasheet S4 in Supplementary Material). Furthermore, two relatively poorly understood clades, the Acidobacteria Gp4 and the TM7, were similarly strongly associated with plant species (Table 1). The primary distinction between the two groups of clades related to strains with known function was that putatively plant symbiosis capable bacteria clades had lower within clade sequence richness than plant-independent clades (mean OTU richness was 93 for plant-dependent versus 411 for plant-independent, two-tailed *t*-test: *p* = 0.005).

#### H3: more remote sites will have fewer but stronger associations between plant species and bacterial clades because facilitation should be more common with increasing environmental harshness

**Figure 5:**
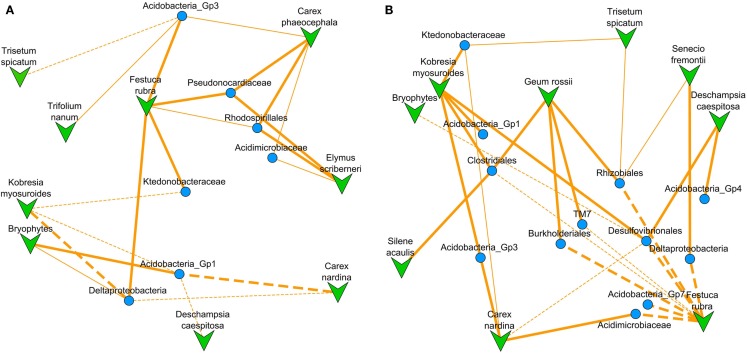
**NMDS ordinated co-occurrence networks based on the correlation matrix after reweighting our clade relative abundances by a harshness metric**. **(A)** For harshness upweighted there were nine plant species and seven bacterial clades which were significantly co-occurring (stress = 0.227). **(B)** For harshness down-weighted there were nine plant species and 12 bacterial clades which were significantly co-occurring (stress = 0.245). Overlaid are lines designating significant model-based interactions between a plant species and bacterial clade with solid for positive and dashed for negative interactions (thin lines represent two-tailed *t*-test for model coefficient significance, *p* < 0.05; thick lines represent significance after Bonferroni correction for multiple comparisons). Because this is an ordination of the correlation matrix, proximity of points to one another represents higher correlation between their abundances across the subnival landscape.

There were only two bacterial clades that had significant models only in the harshness upweighted dataset the Rhodospirillales, and Pseudonocardiaceae (Figure 5A; Datasheet S2 in Supplementary Material; Table 1). The Rhodospirillales also increased its association strength with the dicots *E. scriberneri* and *C. phaeocephala* when environmental harshness was upweighted (Table 1). Similarly, the Actinobacterial clade, Pseudonocardiaceae, increased its association strength with the plants *F. rubrum* and *C. phaeocephala* when harsh sites were upweighted. On the other hand, 7 of the 14 clades had significant model fits only in less harsh sites (sites proximal to the continuous tundra were upweighted; Figure 5B; Datasheet S3 in Supplementary Material; Table 1). Although there were fewer bacterial clades significantly co-occurring with plants in harsh portions of the landscape there was no significant difference in the average correlation strength with plant abundances versus clades preferring less harsh locations (0.42 versus 0.44, two-tailed *t*-test, *p* = 0.60).

Plants species had the greatest number of associations in places where they were most abundant (Table 2). For example, *G. rossii* decreased in abundance with distance from tundra and its correlations with bacterial clades were entirely dependent on associations in sites proximal to the tundra. The number of plant-bacteria associations was lower in harsher portions of the landscape (22 associations near tundra versus 12 near snowline) but the average correlation strength per plant species did not differ between harshness upweighted and downweighted datasets (0.31 versus 0.40; two-tailed *t*-test *p* = 0.42).

## Discussion

This study is the first demonstration, to our knowledge, of plant species and soil bacterial associations across a heterogeneous landscape with a diverse plant community and indicates that plant-bacteria interactions are a key potential driver of the structure of the subnival ecosystem. In fact, 31% of bacterial sequences in this study fell into clades whose relative abundance was significantly correlated to plant species abundance. This percentage represents the presence in bulk soil material of rhizosphere symbiotes, decomposers that grow particularly well on senesced material from the associated plant, and independent bacteria sharing similar environmental preferences (Mahaffee and Kloepper, 1997; Graff and Conrad, 2006). That the percentage of bacteria correlated with plant species abundance in subnival soil is not greater likely reflects the fact that surveys of bulk soil detect a limited percentage of plant-bacteria symbiotes relative to direct analysis of plant roots (Mahaffee and Kloepper, 1997; Graff and Conrad, 2006). Yet it remains to be seen if studies of bulk soils in less extreme landscapes will find similar percentage of their sequences associated with plant species abundances. Importantly, the associations between plant species and bacteria were not evenly distributed across the subnival environment, but showed clear differences in strength and frequency between environmentally harsh sites close to the snowline and the less harsh sites close to continuous tundra (Table 1). The examination of our three hypothesized drivers of community assembly in this landscape (plant abundance, clade potential functional capacity, and environmental harshness) helps place these plant-bacteria co-occurrence patterns for subnival soils in a broader context.

The first of our hypothesized assembly drivers, plant abundance, was thought to influence the distributions of soil bacteria. Although in general plant abundance was not correlated with the number of associated clades in this subnival landscape, the subset of plants that had significant correlations with bacterial clades showed a strong relationship between abundance and mean clade correlation strength as well as number of clades (Figure 4; Table 2). This suggests that abundant plants are good predictors of the bulk soil relative abundance of both generalist plant-dependent bacteria and bacteria directly specializing on the micro-environment created by a plant species. An analogous situation occurs in glacial recession landscapes wherein bacterial diversity increases along with successive plant colonization although only a subset are specifically associated with individual plant species (e.g., Schmidt et al., 2008b; Knelman et al., 2012). While the plant with the greatest number of positive associations, *G. rossii*, was indeed the most abundant plant species, *K. myosuroides* and *C. nardina* had the second highest number of positive associations even though they are only sparsely distributed across this subnival landscape. Interestingly, while *G. rossii* is dominant in moist meadow tundra at Niwot Ridge (Bowman et al., 2004), *K. myosuroides* and *Carex* species are dominant in dry meadow tundra ecotypes (Fisk and Schmidt, 1995; Seastedt and Vaccaro, 2001). Dominance is not the only important factor here, however, as the co-dominant with *G. rossii*, *D. caespitosa*, did not have a large number of associated bacteria. Digging deeper, both *G. rossii* and *K. myosuroides* are known to associate with soil microorganism in oligotrophic conditions (Lipson et al., 2002; Bowman et al., 2004) whereas *D. caespitosa* is thought to prefer higher nutrient soils and may have weaker associations with soil microorganisms (Bowman et al., 2004). Thus, bacterial clades may co-occur with plants not just based on the abundant plants in a specific environment but also based on the plant species’ level of interaction with soil bacteria. This revised hypothesis, that the life history of abundant plants drives association number, is similar to previous observations that bacterial clades associated with plant community types in less extreme environments (e.g., Berg and Smalla, 2009; Eskelinen et al., 2009; Bezemer et al., 2010) and suggests that the signature of plant dominance on co-occurring soil bacterial clades should have transferability across ecosystems.

Clade functional capacity, our second hypothesized driver of assembly, tests the assumption that previously reported functional niche characteristics for bacterial clades predict patterns co-occurrence with plants (i.e., functional niche assembly, Chase and Leibold, 2003). Six of the eight clades that were related to cultured isolates, representing 10% of the sequences in our study, were closely related to strains previously reported to form mutualistic associations with plants; however, these clades did not have a stronger average correlation with associated plants than the other clades in our study (Table 1). However, the two bacterial clades we categorized as likely to be plant-independent were the largest clades in our study (themselves representing 10% of the total sequences) and are known only for their function as generalist soil heterotrophs (Table 1). This difference in diversity between our clades related to previously cultured strains supports the idea that functionally relevant clades (or Operational Taxonomic Units) can be successfully identified using a phylogenetic comparative method to selecting the nodes on a phylogenetic tree of environmental sequences with the greatest correlation to a factor of interest (co-occurrence with plants in this study). Thus, if our assignment of association type is accurate, the strength and likely the specificity of associations may differ within both symbiotic and non-symbiotic soil bacterial guilds; for example, soil heterotrophs have been shown to strongly specialize on a particular plant’s litter or non-specifically attack any detritus (van der Heijden et al., 2008), and symbiotic bacteria have been shown to opportunistically infect a plant but also commonly grow as a free-living form (Rodrigues-Diaz et al., 2008). Therefore, although strength of correlation is not a strong predictor of the type of plant-bacterial associations, clades related to plant-symbiotic bacteria may be more likely to correlate with plant species abundances and with a finer taxonomic resolution than clades related to free-living bacterial species.

An additional note on the potential selection for specific bacterial functions is that only one bacterial clade had only negative significant correlations with plants in this subnival ecosystem – the Desulfovibrionales (1.3% of the total sequences). In the context of correlations, negative interactions could mean either inhibition either from disease and competition or an indirect link via differential response to an environmental gradient. For the subnival ecosystem, the presence of a significant area of exposed soils and microbial crusts selects for bacteria that are adapted to the plant-free state (Nemergut et al., 2007; Freeman et al., 2009) and these crust bacteria should show a negative association with abundant plant species (i.e., fewer plants = more crust bacteria). That so few clades displayed this pattern suggests inhibition between bacteria and plants is rare in the subnival zone, which is in agreement with previous hypotheses about facilitation being the primary driver of species interactions in early successional systems (Chapin et al., 1994; Walker and del Moral, 2003).

Termed environmental harshness, the last of our three hypothesized drivers of subnival soil community assembly is based on the theory that abiotic factors create a selective filter for plant-bacterial associations (Weiher and Keddy, 1995; Diaz et al., 1998; Ackerly, 2003) with fewer but stronger associations under harsher conditions (Callaway and Walker, 1997; Reynolds et al., 2003; Seeds and Bishop, 2009). Within the subnival landscape of Niwot Ridge, there were six significant models in the remote-upweighted dataset whereas there were 12 significant models in the remote-down-weighted dataset. Moreover there were only two bacterial clades that had a significant model fit in only the most environmentally harsh sites (Table 1). This is in agreement with the hypothesis that plant-microbe interactions are less frequent in the more remote portions of the subnival landscape as a result of less plant diversity and low abundance of plant-symbiotic bacteria (Seeds and Bishop, 2009). With these harsh conditions, it is also hypothesized that interactions should be stronger (Reynolds et al., 2003), yet, there was no significant difference in the average correlation strength for clades favoring one end of the harshness gradient or the other. This lower number of harshness upweighted plant-bacteria correlations and similar strength of correlation despite the high correlation of individual plant species with our harshness gradient suggests that environmental filters become more important than biotic filters in harsher environments and is in agreement to what previous work has found at the community scale (Carlson et al., 2012).

Interestingly, both of the bacterial clades that were remote specialists are related to isolates known to garner energy from non-organic sources; members of the Rhodospirillales have demonstrated the ability to perform anaerobic phototrophic sulfate reduction in addition to heterotrophy (Madigan, 2005; Rodrigues-Diaz et al., 2008) and members of the Pseudonocardiaceae have demonstrated the ability to perform hydrogen sulfide oxidation as well as heterotrophy (Reichert et al., 1998; Chen et al., 2009). Because the harshest environmental conditions of the subnival zone are more common in remote portions of the landscape, it may be that these two extremophilic bacterial clades are early colonizers of recently exposed soils and later facilitate plant colonization via their potential to form symbioses or because they improve soil conditions. This phenomenon is similar to what is seen in the primary soils from pyrite weathering (Choler et al., 2001) and experimental work to tease apart the environmental versus biotic association is needed to further explore these patterns.

A caveat with this correlation network approach is that the correlation between a plant species and a bacterial clade may be the result of environmental factors shaping the distribution of both groups independently, a symbiotic/competitive/facilitative interaction, or even a tertiary relationship such as are seen with parasites of a parasite. However, that 75% of the clades to which we could assign hypothetical functions appear to have the potential to form symbioses with plants (six of eight, Table 1; Figure 3) suggests that we are identifying true plant-bacteria associations. It also suggests that the many of the strongly correlated Acidobacteria or TM7 clades, despite their unknown functional capacities, are associated with plants in this landscape.

## Conclusion

We found evidence of three hypothesized assembly drivers between plant species and bulk soil bacteria using a correlational-based analysis along a gradient of environmental harshness in the subnival landscape of Niwot Ridge. First, our findings suggest that the abundance of bacteria-promoting plant species may serve as an indicator or even drive the patterns of plant-associated groups for specific ecosystem types within a landscape. Second, bacterial clades with putative plant symbiosis capacity, as inferred from the close relationship of sequences within a clade to known isolates, were not more likely to be correlated with plant species but have narrower phylogenetic breadth than putative plant-independent clades. Third, our analysis suggests that the influence of environmental harshness on plant-bacterial associations results in fewer plant-bacteria associations in harsh areas than in the less harsh, tundra proximal portion of the landscape. Our results provide the first description of plant-bacteria interactions at the species/clade level and demonstrate an overwhelming number of plant species specific associations with soil bacteria. In addition, facilitative associations appear to be more common than inhibitory associations in the subnival zone. One obvious next step is to test how often these bacteria are indeed found on or within the roots of the plants they co-occur with and if they affect plant growth or survival as opposed to co-occurring due to shared environmental preferences. If co-occurrence relationships are as important for the ecology of high-alpine systems as our correlation network suggests, then the biotic community’s functional resilience is likely sensitive to disturbances affecting either the plant or soil microbial community.

## Conflict of Interest Statement

The authors declare that the research was conducted in the absence of any commercial or financial relationships that could be construed as a potential conflict of interest.

## Supplementary Material

The Supplementary Material for this article can be found online at http://www.frontiersin.org/Terrestrial_Microbiology/10.3389/fmicb.2012.00347/abstract

Supplementary Datasheet S1 **Initial best AIC ranked models predicting bacterial clade relative abundance with plant species abundances**.

Supplementary Datasheet S2 **Best AIC ranked models predicting harshness-upweighted bacterial clade relative abundance with plant species abundances**.

Supplementary Datasheet S3 **Best AIC ranked models predicting harshness-downweighted bacterial clade relative abundance with plant species abundances**.

Supplementary Datasheet S4 **A correlation matrix of plant species abundances and bacterial clade relative abundances (Pearson *r* values)**. A single * designates a correlation that is *p*-value < 0.01 for a two-tailed *t*-test of rho = 0; ** indicates *p* < 0.001; *** indicates *p* < 0.0001. The Bonferroni correction for alpha with 325 comparisons is *p* < 0.0002.

Supplementary Datasheet S5 **A combination of two correlation matrices (Pearson *r* values)**. The upper right triangle in bold represents correlations between bacterial clades and plant abundances with remote locations upweighted. The lower left triangle represents correlations between bacterial clades and plant abundances with remote locations down-weighted. A single * designates a correlation that is *p*-value < 0.01 for a two-tailed *t*-test of rho = 0; ** indicates *p* < 0.001; *** indicates *p* < 0.0001. The Bonferroni correction for alpha with 325 comparisons is *p* < 0.0002.
