# Erratum: Cooccurrence patterns of plants and soil bacteria in the high-alpine subnival zone track environmental harshness

**DOI:** 10.3389/fmicb.2013.00239

**Published:** 2013-08-21

**Authors:** Andrew J. King, Emily C. Farrer, Katharine N. Suding, Steve K. Schmidt

**Affiliations:** ^1^CSIROActon, ACT, Australia; ^2^Department of Environmental Science, Policy and Management, University of California at BerkeleyBerkeley, CA, USA; ^3^Department of Ecology and Evolutionary Biology, University of Colorado, BoulderBoulder, CO, USA

**Keywords:** erratum, community assembly, co-occurrence networks, niwot ridge, plant-microbe interactions

There is an error in the information presented in Table 2. The 11th species should not be listed and the information for the 12th and 13th species in columns 4–6 of the table was incorrectly placed on the species directly above. Table 2 is a summary table and the data miss-attributed are correctly displayed in **Figure 2**.

**Table 2:** **A summary of subnival zone plant species' abundances, number of significant bacterial clade-associations and model fits**.

**Plant species**	**Average (plants/site)**	**SD**	**Positives**	**Negatives**	**Avg str cor (*r*)**	**Correlation with remote index (*r*)**	**Total cor remote upweight**	**Avg corr rm up**	**Total cor remote downweight**	**Avg corr rm down**
*Geum rossii*	8.3	24	4	0	0.44	−0.2	0	0	4	0.60
*Bryophytes*	7.6	13.7	0	0	0	0.05	1	0.52	0	0
*Deschampsia caespitosa*	5.7	14	1	1	0.39	−0.27	0	0	2	0.50
*Trisetum spicatum*	3.9	5.7	0	0	0	0.08	0	0	1	0.42
*Kobresia myosuroides*	3.5	15.3	2	0	0.39	−0.16	1	0.05	5	0.41
*Carex nardina*	3.2	5.7	2	1	0.31	−0.25	1	0.01	2	0.43
*Festuca rubra*	2.9	4	1	0	0.32	0.4	4	0.42	6	0.06
*Trifolium Nanum*	2.7	10.6	0	0	0	−0.04	0	0	0	0
*Senecio fremontii*	2	6.3	1	0	0.26	−0.132	0	0	1	0.43
*Silene acaulis*	1.9	6.1	0	0	0	0.01	0	0	1	0.34
*Elymus scriberneri*	0.9	2.8	1	1	0.25	0.27	2	0.48	0	0
*Carex phaeocephala*	0.9	2.9	2	0	0.31	0.13	3	0.40	0	0

