# Supplementary material for: Co-Occurrence Patterns of Plants and Soil Bacteria in the High-Alpine Subnival Zone Track Environmental Harshness
Source: Front Microbiol. 2012 Oct 11;3:347. doi: 10.3389/fmicb.2012.00347 (PMC3469205; doi:10.3389/fmicb.2012.00347)
Supplement: Supplementary Datasheet S1 — Initial best AIC ranked models predicting bacterial clade relative abundance with plant species abundances. [file 31540_King_DataSheet1.DOC]

**Initial models predicting Bacterial clade relative abundance with plant species abundances.**

**Best AIC Ranked Model: Acidimicrobiaceae**

Call:

lm(formula = species ~ Carex_nardina + Elymus_scriberneri, data = vdmat)

Residuals:

Min 1Q Median 3Q Max

-0.019061 -0.007488 -0.001101 0.005586 0.043541

Coefficients:

Estimate Std. Error t value Pr(>|t|)

(Intercept) 0.0074884 0.0014471 5.175 1.93e-06 ***

Carex_nardina 0.0008902 0.0002112 4.215 7.07e-05 ***

Elymus_scriberneri 0.0007728 0.0004253 1.817 0.0733 .

---

Signif. codes: 0 ‘***’ 0.001 ‘**’ 0.01 ‘*’ 0.05 ‘.’ 0.1 ‘ ’ 1

Residual standard error: 0.01024 on 73 degrees of freedom

Multiple R-squared: 0.2053, Adjusted R-squared: 0.1835

F-statistic: 9.428 on 2 and 73 DF, p-value: 0.000228

**Best AIC Ranked Model: Acidobacteria_Gp1**

Call:

lm(formula = species ~ Carex_nardina + Deschampsia_caespitosa +

Kobresia_myosuroides + Trifolium_Nanum, data = vdmat)

Residuals:

Min 1Q Median 3Q Max

-0.032834 -0.013949 -0.002129 0.010131 0.056223

Coefficients:

Estimate Std. Error t value Pr(>|t|)

(Intercept) 0.0316228 0.0028175 11.224 < 2e-16 ***

Carex_nardina -0.0007147 0.0004035 -1.771 0.08081 .

Deschampsia_caespitosa -0.0004439 0.0001659 -2.676 0.00924 **

Kobresia_myosuroides 0.0005016 0.0001514 3.313 0.00146 **

Trifolium_Nanum -0.0003786 0.0002179 -1.738 0.08660 .

---

Signif. codes: 0 ‘***’ 0.001 ‘**’ 0.01 ‘*’ 0.05 ‘.’ 0.1 ‘ ’ 1

Residual standard error: 0.01983 on 71 degrees of freedom

Multiple R-squared: 0.2546, Adjusted R-squared: 0.2126

F-statistic: 6.063 on 4 and 71 DF, p-value: 0.0002958

**Best AIC Ranked Model: Acidobacteria_Gp3**

Call:

lm(formula = species ~ Carex_nardina + Kobresia_myosuroides +

Silene_acaulis + Trifolium_Nanum + Trisetum_spicatum, data = vdmat)

Residuals:

Min 1Q Median 3Q Max

-0.021179 -0.009022 -0.003257 0.005029 0.031814

Coefficients:

Estimate Std. Error t value Pr(>|t|)

(Intercept) 1.089e-02 2.067e-03 5.268 1.45e-06 ***

Carex_nardina 7.292e-04 2.578e-04 2.828 0.0061 **

Kobresia_myosuroides 2.186e-04 9.671e-05 2.261 0.0269 *

Silene_acaulis -4.403e-04 2.551e-04 -1.726 0.0888 .

Trifolium_Nanum 2.309e-04 1.464e-04 1.577 0.1192

Trisetum_spicatum -5.228e-04 2.593e-04 -2.016 0.0476 *

---

Signif. codes: 0 ‘***’ 0.001 ‘**’ 0.01 ‘*’ 0.05 ‘.’ 0.1 ‘ ’ 1

Residual standard error: 0.01266 on 70 degrees of freedom

Multiple R-squared: 0.2561, Adjusted R-squared: 0.203

F-statistic: 4.821 on 5 and 70 DF, p-value: 0.0007612

**Best AIC Ranked Model: Acidobacteria_Gp4A**

Call:

lm(formula = species ~ Deschampsia_caespitosa + Kobresia_myosuroides,

data = vdmat)

Residuals:

Min 1Q Median 3Q Max

-0.023782 -0.009681 -0.003898 0.005884 0.029748

Coefficients:

Estimate Std. Error t value Pr(>|t|)

(Intercept) 9.681e-03 1.564e-03 6.190 3.18e-08 ***

Deschampsia_caespitosa 5.561e-04 1.032e-04 5.390 8.25e-07 ***

Kobresia_myosuroides -1.788e-04 9.448e-05 -1.893 0.0624 .

---

Signif. codes: 0 ‘***’ 0.001 ‘**’ 0.01 ‘*’ 0.05 ‘.’ 0.1 ‘ ’ 1

Residual standard error: 0.01244 on 73 degrees of freedom

Multiple R-squared: 0.2965, Adjusted R-squared: 0.2772

F-statistic: 15.38 on 2 and 73 DF, p-value: 2.667e-06

**Best AIC Ranked Model: Acidobacteria_Gp7**

Call:

lm(formula = species ~ Carex_nardina + Carex_phaeocephala + Elymus_scriberneri +

Festuca_rubra, data = vdmat)

Residuals:

Min 1Q Median 3Q Max

-0.039029 -0.014979 -0.002308 0.012702 0.096520

Coefficients:

Estimate Std. Error t value Pr(>|t|)

(Intercept) 0.0390286 0.0041182 9.477 3.1e-14 ***

Carex_nardina -0.0009326 0.0005195 -1.795 0.0769 .

Carex_phaeocephala -0.0015244 0.0010056 -1.516 0.1340

Elymus_scriberneri -0.0016676 0.0010825 -1.541 0.1279

Festuca_rubra -0.0018592 0.0007456 -2.494 0.0150 *

---

Signif. codes: 0 ‘***’ 0.001 ‘**’ 0.01 ‘*’ 0.05 ‘.’ 0.1 ‘ ’ 1

Residual standard error: 0.02507 on 71 degrees of freedom

Multiple R-squared: 0.1788, Adjusted R-squared: 0.1325

F-statistic: 3.864 on 4 and 71 DF, p-value: 0.006758

**Best AIC Ranked Model: Burkholderiales**

Call:

lm(formula = species ~ Bryophytes + Deschampsia_caespitosa +

Festuca_rubra + Geum_rossii + Kobresia_myosuroides, data = vdmat)

Residuals:

Min 1Q Median 3Q Max

-0.028490 -0.008901 -0.002164 0.005761 0.045981

Coefficients:

Estimate Std. Error t value Pr(>|t|)

(Intercept) 1.291e-02 2.273e-03 5.680 2.85e-07 ***

Bryophytes 3.132e-04 1.631e-04 1.921 0.0588 .

Deschampsia_caespitosa -1.986e-04 1.175e-04 -1.690 0.0955 .

Festuca_rubra -6.422e-04 4.079e-04 -1.575 0.1198

Geum_rossii 4.194e-04 7.103e-05 5.904 1.15e-07 ***

Kobresia_myosuroides -2.901e-04 1.515e-04 -1.914 0.0597 .

---

Signif. codes: 0 ‘***’ 0.001 ‘**’ 0.01 ‘*’ 0.05 ‘.’ 0.1 ‘ ’ 1

Residual standard error: 0.01386 on 70 degrees of freedom

Multiple R-squared: 0.3561, Adjusted R-squared: 0.3101

F-statistic: 7.744 on 5 and 70 DF, p-value: 7.634e-06

**Best AIC Ranked Model: Clostridiales**

Call:

lm(formula = species ~ Geum_rossii + Kobresia_myosuroides + Silene_acaulis,

data = vdmat)

Residuals:

Min 1Q Median 3Q Max

-0.021360 -0.008704 -0.006164 0.006282 0.080581

Coefficients:

Estimate Std. Error t value Pr(>|t|)

(Intercept) 8.704e-03 1.876e-03 4.640 1.52e-05 ***

Geum_rossii 2.562e-04 7.349e-05 3.486 0.000838 ***

Kobresia_myosuroides 4.491e-04 1.155e-04 3.887 0.000224 ***

Silene_acaulis 4.302e-04 2.802e-04 1.535 0.129049

---

Signif. codes: 0 ‘***’ 0.001 ‘**’ 0.01 ‘*’ 0.05 ‘.’ 0.1 ‘ ’ 1

Residual standard error: 0.01473 on 72 degrees of freedom

Multiple R-squared: 0.3597, Adjusted R-squared: 0.333

F-statistic: 13.48 on 3 and 72 DF, p-value: 4.509e-07

**Best AIC Ranked Model: Deltaproteobacteria**

Call:

lm(formula = species ~ Carex_phaeocephala + Elymus_scriberneri +

Geum_rossii + Senecio_fremontii, data = vdmat)

Residuals:

Min 1Q Median 3Q Max

-0.042754 -0.017510 -0.000497 0.013608 0.065067

Coefficients:

Estimate Std. Error t value Pr(>|t|)

(Intercept) 0.0532803 0.0034005 15.668 < 2e-16 ***

Carex_phaeocephala -0.0016531 0.0009761 -1.694 0.09474 .

Elymus_scriberneri -0.0024396 0.0010030 -2.432 0.01752 *

Geum_rossii -0.0002511 0.0001182 -2.124 0.03716 *

Senecio_fremontii 0.0012916 0.0004514 2.861 0.00554 **

---

Signif. codes: 0 ‘***’ 0.001 ‘**’ 0.01 ‘*’ 0.05 ‘.’ 0.1 ‘ ’ 1

Residual standard error: 0.02447 on 71 degrees of freedom

Multiple R-squared: 0.235, Adjusted R-squared: 0.1919

F-statistic: 5.454 on 4 and 71 DF, p-value: 0.0006916

**Best AIC Ranked Model: Desulfovibrionales**

Call:

lm(formula = species ~ Carex_nardina + Carex_phaeocephala + Elymus_scriberneri +

Trisetum_spicatum, data = vdmat)

Residuals:

Min 1Q Median 3Q Max

-0.0178546 -0.0064483 -0.0008144 0.0047726 0.0292876

Coefficients:

Estimate Std. Error t value Pr(>|t|)

(Intercept) 0.0182026 0.0016425 11.082 < 2e-16 ***

Carex_nardina -0.0006999 0.0002080 -3.365 0.00124 **

Carex_phaeocephala -0.0007328 0.0004022 -1.822 0.07267 .

Elymus_scriberneri -0.0011114 0.0004180 -2.659 0.00967 **

Trisetum_spicatum -0.0003480 0.0002042 -1.704 0.09271 .

---

Signif. codes: 0 ‘***’ 0.001 ‘**’ 0.01 ‘*’ 0.05 ‘.’ 0.1 ‘ ’ 1

Residual standard error: 0.01004 on 71 degrees of freedom

Multiple R-squared: 0.2435, Adjusted R-squared: 0.2009

F-statistic: 5.713 on 4 and 71 DF, p-value: 0.0004808

**Best AIC Ranked Model: Ktedonobacteraceae**

Call:

lm(formula = species ~ Festuca_rubra + Senecio_fremontii + Trisetum_spicatum,

data = vdmat)

Residuals:

Min 1Q Median 3Q Max

-0.07563 -0.03416 -0.01120 0.02933 0.13416

Coefficients:

Estimate Std. Error t value Pr(>|t|)

(Intercept) 0.0340445 0.0077231 4.408 3.57e-05 ***

Festuca_rubra 0.0039383 0.0014471 2.721 0.00814 **

Senecio_fremontii -0.0016525 0.0009368 -1.764 0.08198 .

Trisetum_spicatum 0.0023118 0.0010573 2.186 0.03203 *

---

Signif. codes: 0 ‘***’ 0.001 ‘**’ 0.01 ‘*’ 0.05 ‘.’ 0.1 ‘ ’ 1

Residual standard error: 0.04892 on 72 degrees of freedom

Multiple R-squared: 0.2036, Adjusted R-squared: 0.1704

F-statistic: 6.136 on 3 and 72 DF, p-value: 0.000894

**Best AIC Ranked Model: Pseudonocardiaceae**

Call:

lm(formula = species ~ Carex_phaeocephala + Elymus_scriberneri +

Festuca_rubra, data = vdmat)

Residuals:

Min 1Q Median 3Q Max

-0.023726 -0.007723 -0.005260 0.002303 0.052263

Coefficients:

Estimate Std. Error t value Pr(>|t|)

(Intercept) 0.0052598 0.0022380 2.350 0.02150 *

Carex_phaeocephala 0.0020518 0.0005991 3.425 0.00102 **

Elymus_scriberneri 0.0011830 0.0006372 1.857 0.06745 .

Festuca_rubra 0.0011029 0.0004460 2.473 0.01577 *

---

Signif. codes: 0 ‘***’ 0.001 ‘**’ 0.01 ‘*’ 0.05 ‘.’ 0.1 ‘ ’ 1

Residual standard error: 0.015 on 72 degrees of freedom

Multiple R-squared: 0.2374, Adjusted R-squared: 0.2056

F-statistic: 7.471 on 3 and 72 DF, p-value: 0.000201

**Best AIC Ranked Model: Rhizobiales**

Call:

lm(formula = species ~ Geum_rossii + Senecio_fremontii, data = vdmat)

Residuals:

Min 1Q Median 3Q Max

-0.037246 -0.014464 -0.002528 0.010906 0.081319

Coefficients:

Estimate Std. Error t value Pr(>|t|)

(Intercept) 0.0275579 0.0026742 10.305 6.97e-16 ***

Geum_rossii 0.0004176 0.0001007 4.145 9.04e-05 ***

Senecio_fremontii 0.0008960 0.0003848 2.329 0.0227 *

---

Signif. codes: 0 ‘***’ 0.001 ‘**’ 0.01 ‘*’ 0.05 ‘.’ 0.1 ‘ ’ 1

Residual standard error: 0.02087 on 73 degrees of freedom

Multiple R-squared: 0.2228, Adjusted R-squared: 0.2015

F-statistic: 10.46 on 2 and 73 DF, p-value: 0.0001011

**Best AIC Ranked Model: Rhodospirillales**

Call:

lm(formula = species ~ Bryophytes + Carex_phaeocephala + Elymus_scriberneri +

Geum_rossii + Kobresia_myosuroides + Senecio_fremontii +

Silene_acaulis, data = vdmat)

Residuals:

Min 1Q Median 3Q Max

-0.034748 -0.014217 -0.003667 0.009030 0.061452

Coefficients:

Estimate Std. Error t value Pr(>|t|)

(Intercept) 0.0244911 0.0034690 7.060 1.11e-09 ***

Bryophytes -0.0006046 0.0002527 -2.393 0.01948 *

Carex_phaeocephala 0.0029323 0.0008364 3.506 0.00081 ***

Elymus_scriberneri 0.0026998 0.0008633 3.127 0.00260 **

Geum_rossii -0.0001819 0.0001056 -1.722 0.08963 .

Kobresia_myosuroides 0.0004911 0.0002308 2.128 0.03696 *

Senecio_fremontii -0.0007469 0.0003891 -1.920 0.05911 .

Silene_acaulis -0.0006028 0.0004046 -1.490 0.14088

---

Signif. codes: 0 ‘***’ 0.001 ‘**’ 0.01 ‘*’ 0.05 ‘.’ 0.1 ‘ ’ 1

Residual standard error: 0.02086 on 68 degrees of freedom

Multiple R-squared: 0.3137, Adjusted R-squared: 0.243

F-statistic: 4.44 on 7 and 68 DF, p-value: 0.00041

**Best AIC Ranked Model: TM7**

Call:

lm(formula = species ~ Carex_nardina + Deschampsia_caespitosa +

Elymus_scriberneri + Geum_rossii + Silene_acaulis + Trisetum_spicatum,

data = vdmat)

Residuals:

Min 1Q Median 3Q Max

-0.024088 -0.007108 -0.000203 0.004601 0.038476

Coefficients:

Estimate Std. Error t value Pr(>|t|)

(Intercept) 9.742e-03 1.792e-03 5.436 7.74e-07 ***

Carex_nardina -3.200e-04 2.108e-04 -1.518 0.1335

Deschampsia_caespitosa -1.495e-04 8.658e-05 -1.726 0.0888 .

Elymus_scriberneri -7.062e-04 4.266e-04 -1.655 0.1024

Geum_rossii 2.113e-04 5.023e-05 4.207 7.66e-05 ***

Silene_acaulis -2.989e-04 1.953e-04 -1.531 0.1304

Trisetum_spicatum 3.092e-04 2.074e-04 1.491 0.1406

---

Signif. codes: 0 ‘***’ 0.001 ‘**’ 0.01 ‘*’ 0.05 ‘.’ 0.1 ‘ ’ 1

Residual standard error: 0.01019 on 69 degrees of freedom

Multiple R-squared: 0.2724, Adjusted R-squared: 0.2091

F-statistic: 4.305 on 6 and 69 DF, p-value: 0.0009598
