# Supplementary material for: Co-Occurrence Patterns of Plants and Soil Bacteria in the High-Alpine Subnival Zone Track Environmental Harshness
Source: Front Microbiol. 2012 Oct 11;3:347. doi: 10.3389/fmicb.2012.00347 (PMC3469205; doi:10.3389/fmicb.2012.00347)
Supplement: Supplementary Datasheet S2 — Best AIC ranked models predicting harshness-upweighted bacterial clade relative abundance with plant species abundances. [file 31540_King_DataSheet2.DOC]

**Models predicting harshness upweighted Bacterial clade relative abundance with plant species abundances.**

**Best AIC Ranked Model: Acidimicrobiaceae**

Call:

lm(formula = species ~ Carex_phaeocephala + Elymus_scriberneri +

Festuca_rubra, data = vdmat)

Residuals:

Min 1Q Median 3Q Max

-2.2628 -0.5656 -0.2170 0.5768 3.0548

Coefficients:

Estimate Std. Error t value Pr(>|t|)

(Intercept) 0.47860 0.16445 2.910 0.004801 **

Carex_phaeocephala 0.12362 0.04403 2.808 0.006411 **

Elymus_scriberneri 0.18111 0.04682 3.868 0.000238 ***

Festuca_rubra 0.06454 0.03277 1.969 0.052753 .

---

Signif. codes: 0 ‘***’ 0.001 ‘**’ 0.01 ‘*’ 0.05 ‘.’ 0.1 ‘ ’ 1

Residual standard error: 1.102 on 72 degrees of freedom

Multiple R-squared: 0.2948, Adjusted R-squared: 0.2654

F-statistic: 10.03 on 3 and 72 DF, p-value: 1.325e-05

**Best AIC Ranked Model: Acidobacteria_Gp1**

Call:

lm(formula = species ~ Bryophytes + Carex_nardina + Deschampsia_caespitosa +

Kobresia_myosuroides, data = vdmat)

Residuals:

Min 1Q Median 3Q Max

-6.0454 -2.7841 -0.8479 2.4452 9.1567

Coefficients:

Estimate Std. Error t value Pr(>|t|)

(Intercept) 3.90707 0.53709 7.275 3.67e-10 ***

Bryophytes 0.10691 0.04116 2.597 0.0114 *

Carex_nardina -0.18415 0.07192 -2.560 0.0126 *

Deschampsia_caespitosa -0.06965 0.02940 -2.369 0.0205 *

Kobresia_myosuroides -0.07842 0.03699 -2.120 0.0375 *

---

Signif. codes: 0 ‘***’ 0.001 ‘**’ 0.01 ‘*’ 0.05 ‘.’ 0.1 ‘ ’ 1

Residual standard error: 3.533 on 71 degrees of freedom

Multiple R-squared: 0.2093, Adjusted R-squared: 0.1647

F-statistic: 4.698 on 4 and 71 DF, p-value: 0.00202

**Best AIC Ranked Model: Acidobacteria_Gp3**

Call:

lm(formula = species ~ Carex_phaeocephala + Elymus_scriberneri +

Festuca_rubra + Silene_acaulis + Trifolium_nanum + Trisetum_spicatum,

data = vdmat)

Residuals:

Min 1Q Median 3Q Max

-3.2237 -0.8055 -0.3312 0.2503 5.0529

Coefficients:

Estimate Std. Error t value Pr(>|t|)

(Intercept) 0.80549 0.26504 3.039 0.00335 **

Carex_phaeocephala 0.16189 0.06464 2.504 0.01463 *

Elymus_scriberneri -0.10316 0.07177 -1.437 0.15512

Festuca_rubra 0.14844 0.04941 3.004 0.00371 **

Silene_acaulis -0.05096 0.03249 -1.569 0.12133

Trifolium_nanum 0.05058 0.01956 2.586 0.01182 *

Trisetum_spicatum -0.07413 0.03381 -2.193 0.03169 *

---

Signif. codes: 0 ‘***’ 0.001 ‘**’ 0.01 ‘*’ 0.05 ‘.’ 0.1 ‘ ’ 1

Residual standard error: 1.616 on 69 degrees of freedom

Multiple R-squared: 0.2678, Adjusted R-squared: 0.2041

F-statistic: 4.206 on 6 and 69 DF, p-value: 0.001158

**Best AIC Ranked Model: Acidobacteria_Gp4**

Call:

lm(formula = species ~ Bryophytes + Carex_phaeocephala + Kobresia_myosuroides +

Trifolium_nanum, data = vdmat)

Residuals:

Min 1Q Median 3Q Max

-2.0091 -0.7713 -0.5818 0.5868 6.8575

Coefficients:

Estimate Std. Error t value Pr(>|t|)

(Intercept) 0.73501 0.20951 3.508 0.000787 ***

Bryophytes 0.02645 0.01748 1.513 0.134775

Carex_phaeocephala 0.12948 0.05833 2.220 0.029639 *

Kobresia_myosuroides -0.02811 0.01570 -1.790 0.077657 .

Trifolium_nanum 0.02617 0.01636 1.600 0.113984

---

Signif. codes: 0 ‘***’ 0.001 ‘**’ 0.01 ‘*’ 0.05 ‘.’ 0.1 ‘ ’ 1

Residual standard error: 1.461 on 71 degrees of freedom

Multiple R-squared: 0.1269, Adjusted R-squared: 0.07766

F-statistic: 2.579 on 4 and 71 DF, p-value: 0.04459

**Best AIC Ranked Model: Acidobacteria_Gp7**

Call:

lm(formula = species ~ Bryophytes + Carex_nardina + Kobresia_myosuroides,

data = vdmat)

Residuals:

Min 1Q Median 3Q Max

-3.2433 -1.9211 -0.6071 0.7152 9.7724

Coefficients:

Estimate Std. Error t value Pr(>|t|)

(Intercept) 2.44765 0.40757 6.006 7.08e-08 ***

Bryophytes 0.04538 0.03222 1.408 0.1633

Carex_nardina -0.11186 0.05624 -1.989 0.0505 .

Kobresia_myosuroides -0.04945 0.02894 -1.709 0.0918 .

---

Signif. codes: 0 ‘***’ 0.001 ‘**’ 0.01 ‘*’ 0.05 ‘.’ 0.1 ‘ ’ 1

Residual standard error: 2.768 on 72 degrees of freedom

Multiple R-squared: 0.08276, Adjusted R-squared: 0.04454

F-statistic: 2.165 on 3 and 72 DF, p-value: 0.09952

**Best AIC Ranked Model: Burkholderiales**

Call:

lm(formula = species ~ 1, data = vdmat)

Residuals:

Min 1Q Median 3Q Max

-1.3239 -1.2156 -0.8130 0.6559 7.0281

Coefficients:

Estimate Std. Error t value Pr(>|t|)

(Intercept) 1.3239 0.2048 6.464 9.23e-09 ***

---

Signif. codes: 0 ‘***’ 0.001 ‘**’ 0.01 ‘*’ 0.05 ‘.’ 0.1 ‘ ’ 1

Residual standard error: 1.786 on 75 degrees of freedom

**Best AIC Ranked Model: Clostridiales**

Call:

lm(formula = species ~ Festuca_rubra, data = vdmat)

Residuals:

Min 1Q Median 3Q Max

-2.5579 -0.9827 -0.8411 0.3744 15.7127

Coefficients:

Estimate Std. Error t value Pr(>|t|)

(Intercept) 0.94594 0.35355 2.676 0.00918 **

Festuca_rubra 0.16120 0.07097 2.271 0.02604 *

---

Signif. codes: 0 ‘***’ 0.001 ‘**’ 0.01 ‘*’ 0.05 ‘.’ 0.1 ‘ ’ 1

Residual standard error: 2.485 on 74 degrees of freedom

Multiple R-squared: 0.06517, Adjusted R-squared: 0.05253

F-statistic: 5.159 on 1 and 74 DF, p-value: 0.02604

**Best AIC Ranked Model: Deltaproteobacteria**

Call:

lm(formula = species ~ Bryophytes + Carex_nardina + Deschampsia_caespitosa +

Festuca_rubra + Kobresia_myosuroides, data = vdmat)

Residuals:

Min 1Q Median 3Q Max

-6.3151 -2.7243 -0.6647 1.9240 12.9326

Coefficients:

Estimate Std. Error t value Pr(>|t|)

(Intercept) 4.33266 0.70438 6.151 4.23e-08 ***

Bryophytes 0.10260 0.04785 2.144 0.03551 *

Carex_nardina -0.17149 0.08343 -2.056 0.04356 *

Deschampsia_caespitosa -0.05741 0.03413 -1.682 0.09696 .

Festuca_rubra 0.39359 0.12059 3.264 0.00170 **

Kobresia_myosuroides -0.12844 0.04319 -2.974 0.00403 **

---

Signif. codes: 0 ‘***’ 0.001 ‘**’ 0.01 ‘*’ 0.05 ‘.’ 0.1 ‘ ’ 1

Residual standard error: 4.097 on 70 degrees of freedom

Multiple R-squared: 0.2717, Adjusted R-squared: 0.2197

F-statistic: 5.223 on 5 and 70 DF, p-value: 0.000393

**Best AIC Ranked Model: Desulfovibrionales**

Call:

lm(formula = species ~ Carex_nardina + Elymus_scriberneri + Festuca_rubra +

Kobresia_myosuroides, data = vdmat)

Residuals:

Min 1Q Median 3Q Max

-2.7681 -1.1248 -0.5161 0.4983 6.7009

Coefficients:

Estimate Std. Error t value Pr(>|t|)

(Intercept) 1.60740 0.30064 5.347 1.04e-06 ***

Carex_nardina -0.09510 0.03859 -2.464 0.0162 *

Elymus_scriberneri -0.12599 0.08112 -1.553 0.1249

Festuca_rubra 0.09624 0.05719 1.683 0.0968 .

Kobresia_myosuroides -0.02172 0.01458 -1.490 0.1407

---

Signif. codes: 0 ‘***’ 0.001 ‘**’ 0.01 ‘*’ 0.05 ‘.’ 0.1 ‘ ’ 1

Residual standard error: 1.868 on 71 degrees of freedom

Multiple R-squared: 0.1242, Adjusted R-squared: 0.0749

F-statistic: 2.518 on 4 and 71 DF, p-value: 0.04874

**Best AIC Ranked Model: Ktedonobacteraceae**

Call:

lm(formula = species ~ Bryophytes + Carex_nardina + Deschampsia_caespitosa +

Festuca_rubra + Kobresia_myosuroides, data = vdmat)

Residuals:

Min 1Q Median 3Q Max

-14.943 -5.487 -1.320 4.416 24.552

Coefficients:

Estimate Std. Error t value Pr(>|t|)

(Intercept) 5.12560 1.46193 3.506 0.000798 ***

Bryophytes 0.15562 0.09932 1.567 0.121666

Carex_nardina -0.24437 0.17315 -1.411 0.162590

Deschampsia_caespitosa -0.11327 0.07083 -1.599 0.114274

Festuca_rubra 0.98175 0.25028 3.923 0.000202 ***

Kobresia_myosuroides -0.20373 0.08965 -2.273 0.026125 *

---

Signif. codes: 0 ‘***’ 0.001 ‘**’ 0.01 ‘*’ 0.05 ‘.’ 0.1 ‘ ’ 1

Residual standard error: 8.502 on 70 degrees of freedom

Multiple R-squared: 0.2632, Adjusted R-squared: 0.2105

F-statistic: 5 on 5 and 70 DF, p-value: 0.0005665

**Best AIC Ranked Model: Pseudonocardiaceae**

Call:

lm(formula = species ~ Carex_phaeocephala + Elymus_scriberneri +

Festuca_rubra, data = vdmat)

Residuals:

Min 1Q Median 3Q Max

-4.243 -1.139 -0.179 0.276 9.471

Coefficients:

Estimate Std. Error t value Pr(>|t|)

(Intercept) 0.17897 0.36682 0.488 0.62711

Carex_phaeocephala 0.42979 0.09821 4.376 4.01e-05 ***

Elymus_scriberneri 0.27005 0.10444 2.586 0.01174 *

Festuca_rubra 0.23296 0.07310 3.187 0.00213 **

---

Signif. codes: 0 ‘***’ 0.001 ‘**’ 0.01 ‘*’ 0.05 ‘.’ 0.1 ‘ ’ 1

Residual standard error: 2.459 on 72 degrees of freedom

Multiple R-squared: 0.3466, Adjusted R-squared: 0.3194

F-statistic: 12.73 on 3 and 72 DF, p-value: 9.165e-07

**Best AIC Ranked Model: Rhizobiales**

Call:

lm(formula = species ~ Bryophytes + Elymus_scriberneri + Festuca_rubra +

Kobresia_myosuroides + Silene_acaulis, data = vdmat)

Residuals:

Min 1Q Median 3Q Max

-6.0180 -1.8471 -0.4953 1.1707 10.3355

Coefficients:

Estimate Std. Error t value Pr(>|t|)

(Intercept) 1.90509 0.48211 3.952 0.000183 ***

Bryophytes 0.07916 0.03574 2.215 0.030004 *

Elymus_scriberneri 0.20488 0.12925 1.585 0.117436

Festuca_rubra 0.19537 0.09233 2.116 0.037895 *

Kobresia_myosuroides -0.08238 0.03175 -2.595 0.011527 *

Silene_acaulis 0.08022 0.05807 1.381 0.171528

---

Signif. codes: 0 ‘***’ 0.001 ‘**’ 0.01 ‘*’ 0.05 ‘.’ 0.1 ‘ ’ 1

Residual standard error: 2.996 on 70 degrees of freedom

Multiple R-squared: 0.2004, Adjusted R-squared: 0.1433

F-statistic: 3.51 on 5 and 70 DF, p-value: 0.006886

**Best AIC Ranked Model: Rhodospirillales**

Call:

lm(formula = species ~ Carex_phaeocephala + Elymus_scriberneri +

Festuca_rubra, data = vdmat)

Residuals:

Min 1Q Median 3Q Max

-7.8453 -1.2874 -0.7409 0.6442 11.1537

Coefficients:

Estimate Std. Error t value Pr(>|t|)

(Intercept) 0.90826 0.46125 1.969 0.0528 .

Carex_phaeocephala 0.61236 0.12348 4.959 4.56e-06 ***

Elymus_scriberneri 0.60085 0.13132 4.575 1.93e-05 ***

Festuca_rubra 0.23472 0.09192 2.553 0.0128 *

---

Signif. codes: 0 ‘***’ 0.001 ‘**’ 0.01 ‘*’ 0.05 ‘.’ 0.1 ‘ ’ 1

Residual standard error: 3.092 on 72 degrees of freedom

Multiple R-squared: 0.4353, Adjusted R-squared: 0.4118

F-statistic: 18.5 on 3 and 72 DF, p-value: 5.327e-09

**Best AIC Ranked Model: TM7**

Call:

lm(formula = species ~ Carex_nardina + Carex_phaeocephala + Deschampsia_caespitosa +

Elymus_scriberneri + Festuca_rubra, data = vdmat)

Residuals:

Min 1Q Median 3Q Max

-1.4989 -0.7242 -0.3292 0.3521 3.6803

Coefficients:

Estimate Std. Error t value Pr(>|t|)

(Intercept) 0.779771 0.190977 4.083 0.000116 ***

Carex_nardina -0.038874 0.023018 -1.689 0.095695 .

Carex_phaeocephala 0.075471 0.044684 1.689 0.095672 .

Deschampsia_caespitosa -0.014787 0.009245 -1.599 0.114220

Elymus_scriberneri -0.071131 0.048145 -1.477 0.144047

Festuca_rubra 0.089888 0.033001 2.724 0.008143 **

---

Signif. codes: 0 ‘***’ 0.001 ‘**’ 0.01 ‘*’ 0.05 ‘.’ 0.1 ‘ ’ 1

Residual standard error: 1.11 on 70 degrees of freedom

Multiple R-squared: 0.1808, Adjusted R-squared: 0.1222

F-statistic: 3.089 on 5 and 70 DF, p-value: 0.01411
