# Supplementary material for: Co-Occurrence Patterns of Plants and Soil Bacteria in the High-Alpine Subnival Zone Track Environmental Harshness
Source: Front Microbiol. 2012 Oct 11;3:347. doi: 10.3389/fmicb.2012.00347 (PMC3469205; doi:10.3389/fmicb.2012.00347)
Supplement: Supplementary Datasheet S3 — Best AIC ranked models predicting harshness-downweighted bacterial clade relative abundance with plant species abundances. [file 31540_King_DataSheet3.DOC]

**Models predicting harshness downweighted Bacterial clade relative abundance with plant species abundances.**

**Best AIC Ranked Model: Acidimicrobiaceae**

Call:

lm(formula = species ~ Carex_nardina + Festuca_rubra + Geum_rossii +

Silene_acaulis, data = vdmat)

Residuals:

Min 1Q Median 3Q Max

-3.6284 -1.0870 -0.2402 0.8078 7.0288

Coefficients:

Estimate Std. Error t value Pr(>|t|)

(Intercept) 1.087016 0.270206 4.023 0.000142 ***

Carex_nardina 0.153755 0.033408 4.602 1.78e-05 ***

Festuca_rubra -0.135682 0.047051 -2.884 0.005198 **

Geum_rossii 0.015562 0.007913 1.967 0.053131 .

Silene_acaulis 0.048381 0.031327 1.544 0.126948

---

Signif. codes: 0 ‘***’ 0.001 ‘**’ 0.01 ‘*’ 0.05 ‘.’ 0.1 ‘ ’ 1

Residual standard error: 1.642 on 71 degrees of freedom

Multiple R-squared: 0.34, Adjusted R-squared: 0.3028

F-statistic: 9.142 on 4 and 71 DF, p-value: 5.14e-06

**Best AIC Ranked Model: Acidobacteria_Gp1**

Call:

lm(formula = species ~ Bryophytes + Festuca_rubra + Kobresia_myosuroides +

Senecio_fremontii + Trifolium_nanum, data = vdmat)

Residuals:

Min 1Q Median 3Q Max

-3.5836 -1.5661 -0.5277 0.8587 15.1683

Coefficients:

Estimate Std. Error t value Pr(>|t|)

(Intercept) 2.94240 0.46862 6.279 2.51e-08 ***

Bryophytes -0.06076 0.03421 -1.776 0.080 .

Festuca_rubra -0.12478 0.08441 -1.478 0.144

Kobresia_myosuroides 0.15679 0.03048 5.144 2.34e-06 ***

Senecio_fremontii -0.07379 0.05268 -1.401 0.166

Trifolium_nanum -0.04925 0.03207 -1.536 0.129

---

Signif. codes: 0 ‘***’ 0.001 ‘**’ 0.01 ‘*’ 0.05 ‘.’ 0.1 ‘ ’ 1

Residual standard error: 2.834 on 70 degrees of freedom

Multiple R-squared: 0.3368, Adjusted R-squared: 0.2895

F-statistic: 7.111 on 5 and 70 DF, p-value: 1.987e-05

**Best AIC Ranked Model: Acidobacteria_Gp3**

Call:

lm(formula = species ~ Bryophytes + Carex_nardina + Carex_phaeocephala +

Festuca_rubra + Kobresia_myosuroides + Silene_acaulis, data = vdmat)

Residuals:

Min 1Q Median 3Q Max

-3.2270 -1.0693 -0.6760 0.7556 5.7257

Coefficients:

Estimate Std. Error t value Pr(>|t|)

(Intercept) 1.60132 0.33110 4.836 7.74e-06 ***

Bryophytes -0.03893 0.02250 -1.730 0.08813 .

Carex_nardina 0.11963 0.03868 3.093 0.00286 **

Carex_phaeocephala -0.10829 0.07614 -1.422 0.15948

Festuca_rubra -0.10485 0.05590 -1.876 0.06494 .

Kobresia_myosuroides 0.07739 0.02015 3.840 0.00027 ***

Silene_acaulis -0.05460 0.03667 -1.489 0.14110

---

Signif. codes: 0 ‘***’ 0.001 ‘**’ 0.01 ‘*’ 0.05 ‘.’ 0.1 ‘ ’ 1

Residual standard error: 1.892 on 69 degrees of freedom

Multiple R-squared: 0.3042, Adjusted R-squared: 0.2437

F-statistic: 5.028 on 6 and 69 DF, p-value: 0.0002507

**Best AIC Ranked Model: Acidobacteria_Gp4**

Call:

lm(formula = species ~ Deschampsia_caespitosa + Festuca_rubra +

Senecio_fremontii, data = vdmat)

Residuals:

Min 1Q Median 3Q Max

-3.8365 -1.1847 -0.6512 0.7196 5.7780

Coefficients:

Estimate Std. Error t value Pr(>|t|)

(Intercept) 1.18472 0.29832 3.971 0.000168 ***

Deschampsia_caespitosa 0.09666 0.01664 5.810 1.57e-07 ***

Festuca_rubra -0.10283 0.05559 -1.850 0.068434 .

Senecio_fremontii 0.06925 0.03717 1.863 0.066560 .

---

Signif. codes: 0 ‘***’ 0.001 ‘**’ 0.01 ‘*’ 0.05 ‘.’ 0.1 ‘ ’ 1

Residual standard error: 1.938 on 72 degrees of freedom

Multiple R-squared: 0.4178, Adjusted R-squared: 0.3935

F-statistic: 17.22 on 3 and 72 DF, p-value: 1.572e-08

**Best AIC Ranked Model: Acidobacteria_Gp7**

Call:

lm(formula = species ~ Festuca_rubra, data = vdmat)

Residuals:

Min 1Q Median 3Q Max

-4.996 -2.733 -1.232 1.143 21.224

Coefficients:

Estimate Std. Error t value Pr(>|t|)

(Intercept) 4.9959 0.6823 7.322 2.45e-10 ***

Festuca_rubra -0.4693 0.1370 -3.426 0.001 **

---

Signif. codes: 0 ‘***’ 0.001 ‘**’ 0.01 ‘*’ 0.05 ‘.’ 0.1 ‘ ’ 1

Residual standard error: 4.795 on 74 degrees of freedom

Multiple R-squared: 0.1369, Adjusted R-squared: 0.1253

F-statistic: 11.74 on 1 and 74 DF, p-value: 0.001002

**Best AIC Ranked Model: Burkholderiales**

Call:

lm(formula = species ~ Festuca_rubra + Geum_rossii, data = vdmat)

Residuals:

Min 1Q Median 3Q Max

-6.0577 -1.2844 -0.2759 0.8808 10.4679

Coefficients:

Estimate Std. Error t value Pr(>|t|)

(Intercept) 1.74107 0.34886 4.991 3.95e-06 ***

Festuca_rubra -0.19439 0.06790 -2.863 0.00548 **

Geum_rossii 0.07594 0.01142 6.650 4.61e-09 ***

---

Signif. codes: 0 ‘***’ 0.001 ‘**’ 0.01 ‘*’ 0.05 ‘.’ 0.1 ‘ ’ 1

Residual standard error: 2.375 on 73 degrees of freedom

Multiple R-squared: 0.4112, Adjusted R-squared: 0.3951

F-statistic: 25.49 on 2 and 73 DF, p-value: 4.011e-09

**Best AIC Ranked Model: Clostridiales**

Call:

lm(formula = species ~ Festuca_rubra + Geum_rossii + Kobresia_myosuroides +

Silene_acaulis, data = vdmat)

Residuals:

Min 1Q Median 3Q Max

-2.7585 -0.7633 -0.3022 0.6529 4.0878

Coefficients:

Estimate Std. Error t value Pr(>|t|)

(Intercept) 0.749954 0.234785 3.194 0.00209 **

Festuca_rubra -0.108393 0.045952 -2.359 0.02109 *

Geum_rossii 0.053249 0.007813 6.816 2.55e-09 ***

Kobresia_myosuroides 0.096186 0.012588 7.641 7.74e-11 ***

Silene_acaulis 0.090788 0.029821 3.044 0.00327 **

---

Signif. codes: 0 ‘***’ 0.001 ‘**’ 0.01 ‘*’ 0.05 ‘.’ 0.1 ‘ ’ 1

Residual standard error: 1.565 on 71 degrees of freedom

Multiple R-squared: 0.6853, Adjusted R-squared: 0.6675

F-statistic: 38.65 on 4 and 71 DF, p-value: < 2.2e-16

**Best AIC Ranked Model: Deltaproteobacteria**

Call:

lm(formula = species ~ Carex_phaeocephala + Elymus_scriberneri +

Festuca_rubra + Senecio_fremontii, data = vdmat)

Residuals:

Min 1Q Median 3Q Max

-7.5754 -3.6830 -0.7239 2.3482 15.6160

Coefficients:

Estimate Std. Error t value Pr(>|t|)

(Intercept) 7.02066 0.80083 8.767 6.34e-13 ***

Carex_phaeocephala -0.33003 0.20515 -1.609 0.11211

Elymus_scriberneri -0.34396 0.21839 -1.575 0.11971

Festuca_rubra -0.46648 0.15353 -3.038 0.00333 **

Senecio_fremontii 0.31588 0.09482 3.331 0.00137 **

---

Signif. codes: 0 ‘***’ 0.001 ‘**’ 0.01 ‘*’ 0.05 ‘.’ 0.1 ‘ ’ 1

Residual standard error: 5.134 on 71 degrees of freedom

Multiple R-squared: 0.2987, Adjusted R-squared: 0.2592

F-statistic: 7.559 on 4 and 71 DF, p-value: 3.932e-05

**Best AIC Ranked Model: Desulfovibrionales**

Call:

lm(formula = species ~ Bryophytes + Carex_nardina + Deschampsia_caespitosa +

Elymus_scriberneri + Festuca_rubra + Kobresia_myosuroides,

data = vdmat)

Residuals:

Min 1Q Median 3Q Max

-2.2273 -0.8626 -0.2446 0.5226 4.5028

Coefficients:

Estimate Std. Error t value Pr(>|t|)

(Intercept) 1.94663 0.24691 7.884 3.26e-11 ***

Bryophytes -0.04089 0.01645 -2.485 0.01539 *

Carex_nardina -0.06224 0.02891 -2.153 0.03485 *

Deschampsia_caespitosa 0.03674 0.01167 3.147 0.00243 **

Elymus_scriberneri -0.09257 0.06138 -1.508 0.13606

Festuca_rubra -0.11749 0.04303 -2.730 0.00803 **

Kobresia_myosuroides 0.04010 0.01473 2.722 0.00820 **

---

Signif. codes: 0 ‘***’ 0.001 ‘**’ 0.01 ‘*’ 0.05 ‘.’ 0.1 ‘ ’ 1

Residual standard error: 1.397 on 69 degrees of freedom

Multiple R-squared: 0.3412, Adjusted R-squared: 0.2839

F-statistic: 5.955 on 6 and 69 DF, p-value: 4.703e-05

**Best AIC Ranked Model: Ktedonobacteraceae**

Call:

lm(formula = species ~ Bryophytes + Carex_nardina + Kobresia_myosuroides +

Senecio_fremontii + Trisetum_spicatum, data = vdmat)

Residuals:

Min 1Q Median 3Q Max

-7.5543 -2.6558 -0.7663 1.3166 25.3650

Coefficients:

Estimate Std. Error t value Pr(>|t|)

(Intercept) 2.28519 0.86314 2.648 0.01001 *

Bryophytes -0.11799 0.05967 -1.977 0.05193 .

Carex_nardina 0.20964 0.10307 2.034 0.04575 *

Kobresia_myosuroides 0.24382 0.05349 4.558 2.14e-05 ***

Senecio_fremontii -0.15136 0.09704 -1.560 0.12332

Trisetum_spicatum 0.30896 0.10717 2.883 0.00523 **

---

Signif. codes: 0 ‘***’ 0.001 ‘**’ 0.01 ‘*’ 0.05 ‘.’ 0.1 ‘ ’ 1

Residual standard error: 5.071 on 70 degrees of freedom

Multiple R-squared: 0.3187, Adjusted R-squared: 0.27

F-statistic: 6.549 on 5 and 70 DF, p-value: 4.737e-05

**Best AIC Ranked Model: Pseudonocardiaceae**

Call:

lm(formula = species ~ Bryophytes + Carex_nardina + Kobresia_myosuroides,

data = vdmat)

Residuals:

Min 1Q Median 3Q Max

-1.9176 -0.8432 -0.4335 0.3126 7.2705

Coefficients:

Estimate Std. Error t value Pr(>|t|)

(Intercept) 0.89176 0.23622 3.775 0.000326 ***

Bryophytes -0.03803 0.01867 -2.037 0.045353 *

Carex_nardina 0.04929 0.03259 1.512 0.134866

Kobresia_myosuroides 0.04681 0.01677 2.791 0.006725 **

---

Signif. codes: 0 ‘***’ 0.001 ‘**’ 0.01 ‘*’ 0.05 ‘.’ 0.1 ‘ ’ 1

Residual standard error: 1.604 on 72 degrees of freedom

Multiple R-squared: 0.1169, Adjusted R-squared: 0.08008

F-statistic: 3.176 on 3 and 72 DF, p-value: 0.02916

**Best AIC Ranked Model: Rhizobiales**

Call:

lm(formula = species ~ Carex_phaeocephala + Deschampsia_caespitosa +

Festuca_rubra + Geum_rossii + Kobresia_myosuroides + Senecio_fremontii +

Trisetum_spicatum, data = vdmat)

Residuals:

Min 1Q Median 3Q Max

-5.3102 -2.4977 -0.6793 2.0509 14.2779

Coefficients:

Estimate Std. Error t value Pr(>|t|)

(Intercept) 2.94196 0.60183 4.888 6.51e-06 ***

Carex_phaeocephala -0.21269 0.14031 -1.516 0.13420

Deschampsia_caespitosa 0.05054 0.03128 1.616 0.11079

Festuca_rubra -0.35297 0.10749 -3.284 0.00162 **

Geum_rossii 0.08471 0.01800 4.707 1.28e-05 ***

Kobresia_myosuroides 0.05102 0.02858 1.785 0.07871 .

Senecio_fremontii 0.15238 0.07098 2.147 0.03538 *

Trisetum_spicatum 0.16219 0.07713 2.103 0.03918 *

---

Signif. codes: 0 ‘***’ 0.001 ‘**’ 0.01 ‘*’ 0.05 ‘.’ 0.1 ‘ ’ 1

Residual standard error: 3.498 on 68 degrees of freedom

Multiple R-squared: 0.4714, Adjusted R-squared: 0.417

F-statistic: 8.663 on 7 and 68 DF, p-value: 1.466e-07

**Best AIC Ranked Model: Rhodospirillales**

Call:

lm(formula = species ~ Bryophytes + Carex_nardina + Geum_rossii +

Kobresia_myosuroides + Senecio_fremontii + Silene_acaulis,

data = vdmat)

Residuals:

Min 1Q Median 3Q Max

-3.9597 -1.7530 -0.7046 0.8788 14.4539

Coefficients:

Estimate Std. Error t value Pr(>|t|)

(Intercept) 2.89156 0.49575 5.833 1.6e-07 ***

Bryophytes -0.12335 0.03529 -3.496 0.000831 ***

Carex_nardina 0.08496 0.05987 1.419 0.160403

Geum_rossii -0.02181 0.01487 -1.467 0.146909

Kobresia_myosuroides 0.10181 0.03237 3.145 0.002447 **

Senecio_fremontii -0.08520 0.05472 -1.557 0.124035

Silene_acaulis -0.07782 0.05675 -1.371 0.174724

---

Signif. codes: 0 ‘***’ 0.001 ‘**’ 0.01 ‘*’ 0.05 ‘.’ 0.1 ‘ ’ 1

Residual standard error: 2.933 on 69 degrees of freedom

Multiple R-squared: 0.1957, Adjusted R-squared: 0.1257

F-statistic: 2.798 on 6 and 69 DF, p-value: 0.01704

**Best AIC Ranked Model: TM7**

Call:

lm(formula = species ~ Festuca_rubra + Geum_rossii + Trifolium_nanum,

data = vdmat)

Residuals:

Min 1Q Median 3Q Max

-5.2680 -1.2205 -0.3111 0.7145 7.4767

Coefficients:

Estimate Std. Error t value Pr(>|t|)

(Intercept) 1.253954 0.255378 4.910 5.50e-06 ***

Festuca_rubra -0.119207 0.050101 -2.379 0.020 *

Geum_rossii 0.046079 0.008608 5.353 9.84e-07 ***

Trifolium_nanum -0.032622 0.019634 -1.662 0.101

---

Signif. codes: 0 ‘***’ 0.001 ‘**’ 0.01 ‘*’ 0.05 ‘.’ 0.1 ‘ ’ 1

Residual standard error: 1.736 on 72 degrees of freedom

Multiple R-squared: 0.3253, Adjusted R-squared: 0.2972

F-statistic: 11.57 on 3 and 72 DF, p-value: 2.829e-06
